# Developing an Evidence-Informed Strategic Framework for Veterinary Vaccine Manufacturing Modernization Toward PIC/S GMP Compliance

**DOI:** 10.3390/vaccines14090738

**Published:** 2026-08-26

**Authors:** Terdsak Yano, Thanaporn Eiamsam-ang, Tossapond Kewprasopsak, Panuwat Yamsakul

**Affiliations:** 1 Department of Veterinary Medicine, Faculty of Veterinary Medicine, Chiang Mai University, Chiang Mai 50200, Thailand; terdsak.yano@cmu.ac.th (T.Y.); thanaporn.e@cmu.ac.th (T.E.-a.); 2 School of Economics, Faculty of Economics, Chiang Mai University, Chiang Mai 50200, Thailand; tossapond.kew@cmu.ac.th

**Keywords:** evidence-informed framework, framework development methodology, implementation science, PIC/S GMP, veterinary vaccine manufacturing, knowledge translation, regulatory science, systems thinking

## Abstract

**Background/Objectives**: Veterinary vaccine manufacturing is essential for controlling transboundary animal diseases, strengthening animal health systems, and supporting livestock development, particularly in Southeast Asia where livestock production continues to expand. However, modernization toward Pharmaceutical Inspection Co-operation Scheme (PIC/S) Good Manufacturing Practice (GMP) compliance remains challenging because it requires coordinated development across regulatory, technical, organizational, financial, and governance domains. This study aimed to develop an evidence-informed strategic framework for veterinary vaccine manufacturing modernization through a structured and systematically documented framework development methodology integrating multidisciplinary evidence and practice-informed implementation experience. **Methods**: An evidence-informed framework development study was conducted through the multidisciplinary integration of multiple evidence sources, including international regulatory guidance, peer-reviewed scientific literature, multidisciplinary feasibility assessment, and practice-informed implementation experience derived from a government-commissioned consultancy project. Framework development followed a structured methodology comprising evidence collection, evidence categorization, multidisciplinary evidence synthesis, framework development, iterative refinement, and expert-informed validation. Only generalized methodological principles and potentially transferable implementation knowledge were incorporated to ensure confidentiality of project-specific information. **Results**: Multidisciplinary evidence synthesis identified ten evidence domains encompassing regulatory, technical, organizational, economic, legal, environmental, governance, workforce, infrastructure, and implementation considerations. These domains were translated through a structured process into an evidence-informed strategic framework organized around four sequential modernization phases—Design, Construction, Validation, and Continuous Improvement—supported by cross-cutting strategic enablers. Iterative refinement through expert review, technical consultation, and implementation verification strengthened the framework’s scientific consistency, regulatory alignment, and practical applicability. **Conclusions**: Veterinary vaccine manufacturing modernization should be regarded as a multidisciplinary organizational transformation rather than solely as an infrastructure or engineering project. Beyond proposing a strategic framework, this study provides a transparent, systematically documented and traceable methodology for translating multidisciplinary evidence into strategic decision-support tools. The proposed framework may assist governments, regulatory authorities, vaccine manufacturers, and researchers in developing evidence-informed modernization strategies for veterinary biological manufacturing systems, particularly in low- and middle-income countries, subject to adaptation to specific regulatory, technical, institutional, and resource contexts.

## 1. Introduction

The livestock sector plays a fundamental role in global food security, economic development, and rural livelihoods, particularly in developing countries where livestock production continues to expand in response to increasing demand for animal-derived protein [1,2]. However, the increasing frequency and geographic spread of transboundary animal diseases (TADs) have become major threats to sustainable livestock production, international trade, and global food security. Diseases such as foot-and-mouth disease (FMD), African swine fever (ASF), classical swine fever (CSF), lumpy skin disease (LSD), and peste des petits ruminants (PPR) continue to cause substantial economic losses through reduced productivity, trade restrictions, and high animal mortality [3,4,5,6]. Vaccination remains one of the most effective and cost-efficient interventions for preventing and controlling these diseases, reducing their economic impact, and strengthening national and regional animal health systems [7]. Simultaneously, rapid urbanization, population growth, and increasing household incomes are driving continued expansion of livestock production across many developing regions, particularly in Southeast Asia, resulting in a growing demand for safe, effective, and affordable veterinary vaccines [2]. Consequently, strengthening veterinary vaccine manufacturing capacity has become a strategic priority for enhancing disease preparedness, livestock productivity, food security, and regional biosecurity resilience [8].

Despite the growing demand for veterinary vaccines, establishing and modernizing vaccine manufacturing facilities remains one of the most technically demanding and resource-intensive challenges in the animal health sector. Unlike conventional pharmaceutical manufacturing, vaccine production involves complex biological processes that require highly specialized infrastructure, stringent biosafety measures, qualified personnel, comprehensive quality management systems, and continuous regulatory oversight [8,9]. Achieving compliance with internationally recognized Good Manufacturing Practice (GMP) standards, particularly those established by the Pharmaceutical Inspection Co-operation Scheme (PIC/S), further increases the complexity of facility development by requiring risk-based quality management, validated manufacturing processes, contamination control, data integrity, and lifecycle quality assurance [10,11]. These requirements present considerable challenges for manufacturers in low- and middle-income countries, where infrastructure limitations, workforce shortages, constrained financial resources, and evolving regulatory systems often impede sustainable vaccine manufacturing capacity [9,12,13].

Although substantial progress has been made in veterinary vaccine manufacturing research, existing studies have largely addressed individual components of manufacturing systems rather than their integration into a comprehensive modernization strategy. Previous investigations have predominantly focused on specific topics, including vaccine production technologies, biosafety and biocontainment, Good Manufacturing Practice (GMP) compliance, regulatory frameworks, technology transfer, quality management systems, or economic and investment analyses [8,9,11,14]. While these studies have significantly advanced knowledge within their respective disciplines, they often examine these domains independently and provide limited guidance for coordinating technical, regulatory, organizational, financial, and strategic decision-making during large-scale vaccine manufacturing modernization projects. Consequently, decision-makers frequently encounter challenges in translating fragmented evidence into practical implementation strategies, particularly in resource-constrained settings where infrastructure development, regulatory strengthening, investment planning, and institutional capacity must be addressed simultaneously.

In practice, however, modernization of veterinary vaccine manufacturing facilities extends far beyond facility renovation or engineering design. Real-world modernization projects require simultaneous consideration of multiple interdependent domains, including technical feasibility, regulatory compliance, economic viability, legal and intellectual property requirements, environmental sustainability, organizational capacity, stakeholder engagement, governance, and long-term investment strategies. Decisions made within one domain frequently influence outcomes in others, creating complex interrelationships that cannot be adequately addressed through discipline-specific approaches alone [10,11]. Consequently, successful modernization requires a systems-based planning process capable of integrating scientific evidence, regulatory requirements, institutional priorities, and implementation feasibility into a coherent decision-making framework. Despite the practical importance of this multidisciplinary perspective, it remains insufficiently represented in the current veterinary vaccine manufacturing literature, where most studies continue to examine individual components of modernization in isolation [8,15].

Although growing attention has been directed toward strengthening veterinary vaccine manufacturing capacity, published studies describing how multidisciplinary evidence can be systematically translated into strategic decision-making frameworks remain limited. To the best of our knowledge, few studies have reported framework development approaches that explicitly integrate multidisciplinary feasibility assessment with practical implementation experience to support vaccine manufacturing modernization. Existing publications generally provide recommendations within individual disciplines but rarely describe how technical, regulatory, economic, legal, environmental, organizational, and governance considerations can be synthesized into a transferable framework for planning and implementation. Consequently, there remains a critical need for an evidence-informed and practice-informed framework capable of supporting strategic decision-making while remaining adaptable to different institutional, regulatory, and economic contexts, particularly in developing countries [14].

The objective of this study was not only to develop an evidence-informed strategic framework for veterinary vaccine manufacturing modernization toward PIC/S GMP compliance, but also to demonstrate a transparent and transferable framework development methodology that can be adapted to support similar modernization initiatives in other resource-constrained settings. The proposed framework was informed through the integration of international regulatory evidence, multidisciplinary feasibility assessment, and practical implementation experience derived from a real-world vaccine manufacturing modernization project. Rather than presenting project-specific operational findings, this study synthesizes transferable methodological insights and strategic planning principles that can support decision-making for veterinary vaccine manufacturing modernization in resource-constrained settings. The framework is intended to assist policymakers, regulatory authorities, public institutions, and industry stakeholders in designing sustainable modernization strategies that are scientifically robust, operationally feasible, and adaptable to diverse institutional and regulatory contexts [8,9,10,11].

## 2. Materials and Methods

### 2.1. Study Design

This study employed an evidence-informed framework development methodology to establish a practical implementation roadmap for upgrading veterinary vaccine manufacturing facilities toward compliance with Pharmaceutical Inspection Co-operation Scheme Good Manufacturing Practice (PIC/S GMP) requirements. Rather than evaluating a single manufacturing facility, the study aimed to develop a transferable strategic framework that integrates scientific evidence, regulatory requirements, multidisciplinary feasibility assessment, and stakeholder perspectives into a structured implementation model.

Framework development followed an iterative process consisting of evidence collection, multidisciplinary feasibility assessment, stakeholder consultation, evidence synthesis, framework construction, and expert refinement. Information was integrated through a structured process from international regulatory guidance, scientific literature, technical implementation experience obtained during a national consultancy project, and structured stakeholder consultations involving government agencies, veterinary professionals, industry representatives, academic experts, and livestock producers. Stakeholder engagement was incorporated to ensure that the proposed framework reflected both regulatory expectations and practical implementation needs across the veterinary vaccine manufacturing sector.

**Figure 1:**
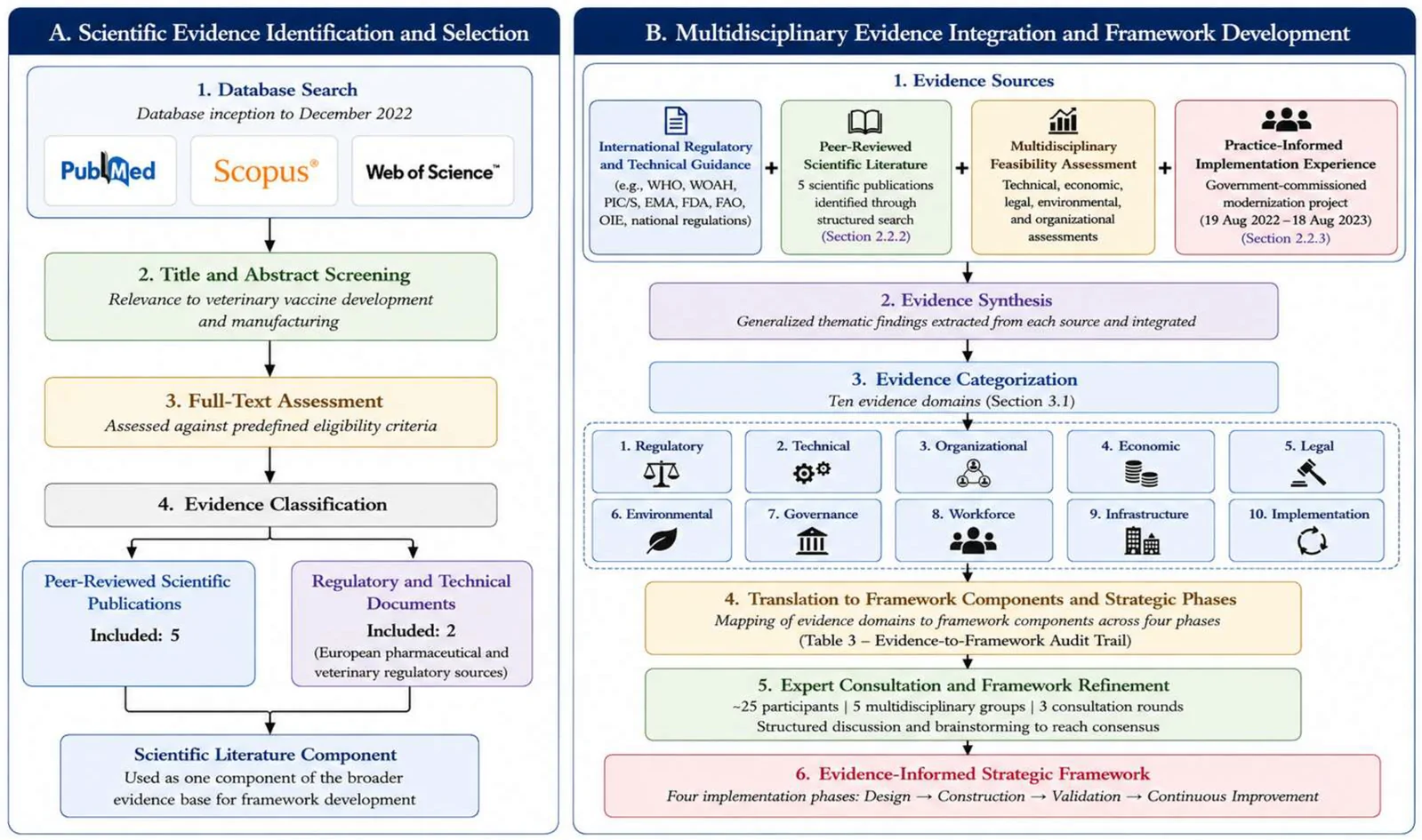
Evidence identification, integration, and framework development process. (**A**) Scientific evidence identification and selection for the literature component, including database searching, relevance screening, full-text assessment, and evidence classification. (**B**) Multidisciplinary integration of regulatory and technical guidance, scientific literature, feasibility assessment, and practice-informed implementation experience, followed by evidence synthesis, categorization into ten evidence domains, translation into framework components, and multidisciplinary consultation and refinement leading to the final evidence-informed strategic framework.

The resulting framework was designed to support strategic decision-making for pharmaceutical quality system modernization by addressing technical, regulatory, economic, legal, environmental, organizational, and implementation dimensions simultaneously. The overall study design is illustrated in Figure 1.

### 2.2. Sources of Evidence

The proposed framework was developed through the multidisciplinary integration of multiple complementary sources of evidence. Rather than relying solely on scientific literature, this study adopted an evidence-informed approach by incorporating international regulatory guidance, peer-reviewed scientific publications, multidisciplinary feasibility assessment, and practice-informed implementation experience. These diverse evidence sources were combined to ensure that the proposed framework was scientifically rigorous, practically applicable, and aligned with internationally recognized regulatory standards.

The evidence sources were categorized into four complementary domains: (i) international regulatory and technical documents, (ii) peer-reviewed scientific literature, (iii) multidisciplinary feasibility assessment conducted during a national veterinary vaccine manufacturing modernization project, and (iv) practice-informed implementation experience. Each evidence source contributed distinct perspectives to framework development, allowing scientific evidence to be interpreted within practical implementation and policy contexts.

#### 2.2.1. International Regulatory Documents

International regulatory documents were reviewed to establish the regulatory principles and quality requirements that underpin veterinary vaccine manufacturing modernization. Primary references included the Pharmaceutical Inspection Co-operation Scheme (PIC/S) Guide to Good Manufacturing Practice (GMP) for Medicinal Products, the World Health Organization (WHO) Good Manufacturing Practices for Biological Products, relevant guidance from the European Medicines Agency (EMA), and selected technical guidance issued by the United States Food and Drug Administration (US FDA). These documents were used to identify internationally accepted requirements for pharmaceutical quality systems, facility design, manufacturing operations, contamination control, quality risk management, process validation, documentation, and quality assurance [10,11,16,17].

The regulatory review focused on extracting common regulatory principles applicable to veterinary biological manufacturing rather than comparing individual regulatory agencies. These principles served as the regulatory foundation for subsequent framework development and evidence synthesis.

#### 2.2.2. Peer-Reviewed Scientific Literature

Peer-reviewed scientific literature was reviewed to establish the scientific foundation supporting veterinary vaccine manufacturing modernization and to complement international regulatory and technical guidance. The literature component was conducted as a structured evidence search to support framework development rather than as a systematic review, scoping review, or meta-analysis. Accordingly, the purpose of the search was to identify scientific evidence and transferable concepts relevant to veterinary vaccine development, manufacturing, quality systems, biosafety, and implementation rather than to quantitatively estimate an intervention effect [18].

The literature search was conducted in PubMed, Scopus, and Web of Science and covered the period from database inception to December 2022. Search terms were used individually and in combination and included “veterinary vaccine”, “veterinary vaccine development”, “veterinary vaccine manufacturing”, “veterinary biologicals”, “vaccine production”, “vaccine manufacturing”, “Good Manufacturing Practice”, “GMP”, “PIC/S”, “pharmaceutical quality system”, “biosafety”, “biocontainment”, “quality management”, “facility modernization”, and “vaccine implementation”. Boolean operators (AND/OR) were used where appropriate to combine vaccine-related terms with manufacturing, regulatory, quality, and biosafety concepts. Reference lists of relevant publications were also manually screened to identify additional potentially relevant sources.

Publications were considered eligible when they provided information directly relevant to one or more aspects of veterinary vaccine development or manufacturing, including vaccine production technologies, biological-product manufacturing, quality and regulatory requirements, biosafety and biocontainment, manufacturing processes, or considerations applicable to modernization of veterinary vaccine production systems. Priority was given to studies and reviews with direct relevance to veterinary vaccines or biological manufacturing. Publications were excluded when they did not provide information relevant to vaccine development, manufacturing, quality, regulatory, biosafety, or implementation considerations within the scope of framework development.

Following relevance screening and full-text assessment, five peer-reviewed scientific publications were retained as the core scientific literature informing this component of the framework development [19,20,21,22,23]. Two additional European regulatory sources identified during the evidence-gathering process were classified separately under international regulatory and technical documents (Section 2.2.1) [24,25] and were not counted among the five peer-reviewed publications. Thus, this focused literature-screening pathway yielded five peer-reviewed publications and two additional regulatory sources, while the broader regulatory evidence base described in Section 2.2.1 was identified separately. Rather than extracting quantitative effect estimates, relevant information was examined for concepts, technical considerations, implementation issues, and transferable principles that could contribute to subsequent multidisciplinary evidence synthesis.

Importantly, the scientific literature represented only one component of the evidence base. The ten evidence domains and subsequent strategic framework were therefore not derived from these publications alone but from their integration with international regulatory and technical guidance, multidisciplinary feasibility assessment, and practice-informed implementation experience, as summarized in Table 1.

#### 2.2.3. Practice-Informed Evidence

Practice-informed evidence was derived from a government-commissioned feasibility and strategic planning project for the modernization of an established veterinary vaccine manufacturing system toward alignment with PIC/S GMP requirements. The project was conducted over a one-year period, from 19 August 2022 to 18 August 2023, and provided the practical implementation context for examining how regulatory, technical, infrastructure, organizational, economic, and governance considerations interact during veterinary vaccine manufacturing modernization.

To provide sufficient contextual information while maintaining contractual confidentiality, the project setting is described here in a de-identified and generalized manner. The assessment involved a large-scale veterinary biological manufacturing system comprising four production facilities supporting vaccine production for major livestock sectors, including poultry, swine, and ruminants. The manufacturing portfolio covered multiple veterinary vaccine types, including vaccines against infectious bronchitis, infectious bursal disease, Newcastle disease, duck plague, classical swine fever, foot-and-mouth disease, and hemorrhagic septicemia. These products represented different manufacturing and biosafety requirements and therefore provided a practical setting in which modernization requirements could be considered across multiple vaccine-production contexts.

At baseline, the manufacturing system represented an established public-sector veterinary vaccine production capacity primarily intended to support domestic animal-disease prevention and control. The modernization assessment examined the feasibility and strategic requirements for transitioning the existing manufacturing system toward PIC/S GMP-aligned production, including regulatory compliance, facility and engineering requirements, biosafety and biosecurity, quality systems, workforce capability, investment feasibility, organizational readiness, and long-term operational sustainability. The intended strategic end-state was not limited to physical facility upgrading but encompassed development of a sustainable manufacturing system capable of meeting internationally recognized GMP expectations and supporting future veterinary vaccine production capacity in Thailand and, where appropriate, the broader Southeast Asian context.

Practice-informed evidence was abstracted at the level of generalizable modernization considerations rather than institution-specific findings. Project observations and implementation considerations were examined for recurring issues affecting modernization planning and were subsequently integrated with regulatory and scientific evidence during framework development. No facility-specific production capacities, engineering drawings, detailed process configurations, financial estimates, proprietary manufacturing information, or other information that could identify the commissioning organization or disclose protected project findings were included in the analysis presented in this manuscript.

Accordingly, the project served as a practice-informed evidence source for framework development rather than as a case study whose institutional results were reported directly. This approach allowed practical implementation experience to inform the framework while preserving the confidentiality and intellectual property obligations associated with the government-commissioned project.

#### 2.2.4. Implementation Experience and Expert Knowledge Integration

Beyond scientific and regulatory evidence, framework development was informed by implementation experience gained during the execution of a multidisciplinary veterinary vaccine manufacturing modernization project. Rather than relying exclusively on published literature, the framework was refined through continuous interaction between evidence synthesis and practical implementation activities undertaken throughout the project. This iterative process enabled scientific knowledge to be interpreted within operational contexts and facilitated identification of practical considerations that are seldom described in academic publications.

Implementation experience contributed to the refinement of framework components related to strategic planning, implementation sequencing, multidisciplinary coordination, regulatory readiness, organizational preparedness, and long-term sustainability. These practical insights were incorporated only when they could be generalized beyond the specific project context and were supported by scientific evidence and internationally recognized regulatory principles. No confidential operational information, institution-specific findings, or proprietary project data were included in the framework development process.

Accordingly, the proposed framework represents the integration of published scientific evidence, international regulatory guidance, and transferable implementation knowledge generated during real-world modernization activities. This approach was adopted to enhance the practical applicability of the framework while maintaining scientific rigor, methodological transparency, and compliance with contractual confidentiality obligations [26].

### 2.3. Framework Development Methodology

The strategic framework was developed through a structured, iterative, and evidence-informed methodology designed to integrate multiple sources of scientific, regulatory, and practice-based evidence into a coherent implementation framework [10,11,26,27]. Framework development was conducted through six sequential but iterative steps. Each step had a distinct methodological function, progressing from evidence identification to validation of the final strategic framework (Figure 1).

#### 2.3.1. Step 1: Evidence Collection

Evidence was collected from the four sources described in Section 2.2: international regulatory and technical documents, peer-reviewed scientific literature, multidisciplinary feasibility assessment, and practice-informed implementation experience. Evidence relevant to veterinary vaccine manufacturing modernization, PIC/S GMP alignment, technical feasibility, organizational capability, and implementation was retained for subsequent synthesis.

#### 2.3.2. Step 2: Evidence Categorization

Relevant evidence was categorized according to recurring regulatory, technical, organizational, economic, legal, environmental, governance, workforce, infrastructure, and implementation considerations. Categorization provided the initial structure for identifying relationships across otherwise distinct evidence sources.

#### 2.3.3. Step 3: Multidisciplinary Synthesis

Evidence within and across categories was compared to identify convergent, complementary, and interdependent considerations. Particular attention was given to interactions among regulatory requirements, vaccine-production processes, biosafety and biocontainment, infrastructure, workforce capability, economic feasibility, and institutional governance.

#### 2.3.4. Step 4: Framework Construction

The synthesized evidence domains were translated into operational framework components and mapped to the modernization pathway. Components were organized according to their primary function and relationship with the four strategic phases: Design, Construction, Validation, and Continuous Improvement.

#### 2.3.5. Step 5: Framework Refinement

The preliminary framework was iteratively refined to improve the relationships among framework components, remove duplication, and ensure that veterinary vaccine-specific technical requirements were appropriately represented. Refinement also considered practical feasibility and implementation sequencing derived from the government-commissioned project.

#### 2.3.6. Step 6: Framework Validation

The refined framework underwent multidisciplinary technical and expert review to assess regulatory relevance, technical coherence, feasibility, and practical applicability. Detailed procedures for participant selection, consultation rounds, and consensus-based refinement are described in Section 2.5.

### 2.4. Evidence Integration and Framework Synthesis

**Figure 2:**
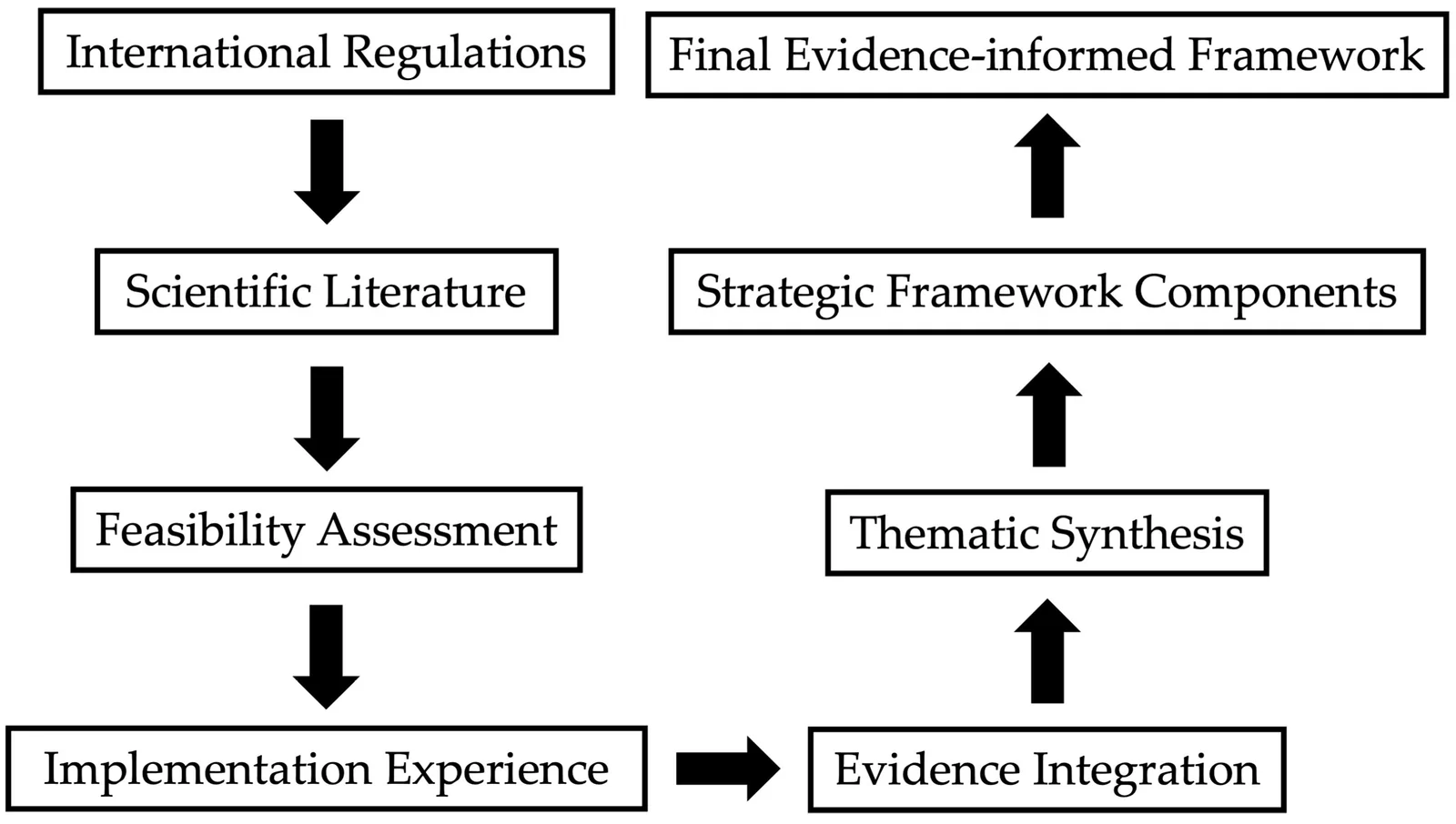
Evidence integration process used for framework synthesis.

Evidence integration was conducted using a thematic synthesis approach to combine scientific evidence, international regulatory requirements, multidisciplinary feasibility assessment, and implementation experience into a unified conceptual framework (Figure 2). Rather than considering each evidence source independently, the study employed an iterative process to identify complementary relationships, shared implementation principles, and cross-disciplinary interactions among the four evidence domains.

Regulatory guidance provided the quality standards and compliance requirements necessary for pharmaceutical manufacturing. Scientific literature contributed current knowledge, best practices, and implementation concepts reported in previous studies. Multidisciplinary feasibility assessment provided contextual understanding of technical, organizational, economic, legal, environmental, and governance considerations relevant to vaccine manufacturing modernization. Practice-based implementation experience enabled these diverse sources of evidence to be interpreted within real-world operational settings and facilitated translation of theoretical knowledge into practical implementation strategies.

Following thematic synthesis, evidence from the four domains was consolidated into a series of strategic implementation components representing the essential dimensions of veterinary vaccine manufacturing modernization. Evidence was incorporated into the framework only when supported by scientific literature, aligned with internationally recognized regulatory principles, and considered operationally feasible based on multidisciplinary assessment and implementation experience. This triangulation process was applied to enhance scientific credibility, practical relevance, and transferability of the proposed framework [28,29,30].

To illustrate how practice-informed evidence contributed to framework development without disclosing confidential project information, a generalized application pathway was constructed from the modernization experience. In this example, an established veterinary vaccine manufacturing system seeking alignment with PIC/S GMP requirements would first undergo assessment of its existing product portfolio, regulatory requirements, manufacturing processes, facility configuration, biosafety requirements, quality systems, workforce capability, and organizational readiness. Identified requirements would then be categorized across the relevant evidence domains rather than being treated as isolated engineering deficiencies.

For example, a requirement affecting a vaccine-production area may simultaneously involve regulatory compliance, biosafety containment, facility design, utilities, process segregation, personnel competency, quality-system requirements, investment feasibility, and implementation sequencing. The framework therefore requires these interdependent considerations to be evaluated collectively during the Design phase before major infrastructure decisions are made. The resulting priorities subsequently inform the Construction phase, followed by qualification and validation activities during the Validation phase, and are maintained through quality management, workforce development, regulatory monitoring, and performance review during Continuous Improvement.

This generalized pathway illustrates how field-based implementation experience informed the transition from individual technical observations to multidisciplinary evidence domains and, subsequently, to the phased strategic framework. No project-specific facility characteristics, assessment findings, investment values, or confidential implementation decisions are reproduced in this example.

To improve traceability between evidence identification and framework construction, an evidence-to-framework audit trail was developed. Evidence from regulatory documents, scientific literature, multidisciplinary feasibility assessment, and practice-informed implementation experience was first summarized as generalized thematic findings. These findings were then categorized within the ten evidence domains and mapped to corresponding framework components and implementation phases. The resulting evidence-to-framework mapping is presented in Table 2. This process enabled the conceptual pathway from source evidence to the final strategic framework to be documented without disclosing confidential project-specific information.

### 2.5. Framework Validation and Expert Review

The preliminary framework was subjected to multidisciplinary expert review and technical consultation to assess its regulatory relevance, technical coherence, practical applicability, and feasibility for veterinary vaccine manufacturing modernization. These activities formed part of the technical review and decision-support process associated with framework refinement rather than a qualitative research process designed to collect individual opinions or generate human-participant research data.

Approximately 25 participants representing five multidisciplinary groups contributed to the consultation and framework refinement process. Participants were selected purposively on the basis of their professional expertise, institutional responsibilities, or direct involvement in relevant components of the veterinary vaccine development, manufacturing, regulatory, economic, and implementation pathway. The five groups broadly represented: (i) academic and veterinary specialists with expertise relevant to animal species and vaccine production; (ii) GMP/PIC/S, pharmaceutical quality-system, and related technical specialists; (iii) economic, investment, and feasibility specialists; (iv) governmental and policy-level representatives involved in animal-health and vaccine-related decision-making; and (v) private-sector and implementation representatives with relevant operational experience.

Framework review was conducted through three consultation rounds. The first round focused primarily on the completeness and relevance of the evidence domains and the major regulatory, technical, organizational, economic, and implementation considerations. The second round examined the translation of these domains into framework components and their practical relationships within the modernization pathway. The third round reviewed the overall framework structure, implementation sequence, and practical applicability, including the progression through the Design, Construction, Validation, and Continuous Improvement phases.

The consultation process used structured multidisciplinary discussion and brainstorming rather than a formal Delphi procedure or quantitative expert-rating method. Where differing perspectives arose, the issues were discussed collectively with reference to applicable regulatory requirements, scientific and technical evidence, feasibility considerations, and practical implementation constraints. Consensus was defined operationally as the absence of substantive unresolved objection to the proposed framework component or its placement after multidisciplinary discussion. Where agreement was not initially reached, the relevant component was revised and reconsidered during the consultation process until a practically acceptable position was reached [10,11,28,29].

No statistical analysis was applied to the consultation process because expert contributions were used for technical verification, feasibility-oriented review, and framework refinement, rather than for quantitative measurement of expert agreement. The final framework therefore reflects convergence among regulatory, technical, economic, organizational, policy, and implementation considerations while retaining consistency with the evidence sources described in Section 2.2.

To preserve confidentiality, individual participants, institutional affiliations, meeting records, and project-specific recommendations are not reported. Only generalized methodological information concerning the composition, purpose, and process of the consultation is presented.

### 2.6. Ethical, Confidentiality, and Data Governance Considerations

The practice-informed component of this study originated from a government-commissioned technical feasibility and strategic planning project. The multidisciplinary consultations described in Section 2.5 were conducted as part of the project’s technical review, feasibility assessment, and decision-support activities. They were not designed as interviews, focus groups, surveys, or other forms of qualitative human-subject research, and no individual-level responses, personal data, or identifiable participant information were collected or analyzed for the present study.

The manuscript does not report individual opinions or attribute statements to any consultation participant. Information derived from the consultation process was used only at an aggregated and generalized level to support technical verification and refinement of the framework. Participation in the project consultations occurred within the professional and institutional roles of the individuals involved in the commissioned technical process.

The government-commissioned project was subject to contractual confidentiality and intellectual-property requirements. Accordingly, facility-identifying information, proprietary manufacturing data, detailed engineering information, financial analyses, project-specific recommendations, and identifiable consultation records were excluded from the manuscript. Only generalized and transferable methodological and strategic insights are presented.

The study did not involve experimental interventions in humans or animals, and the manuscript does not analyze personal or identifiable human-participant data. Ethical and confidentiality considerations were therefore addressed through the governance arrangements of the commissioned project and through exclusion of identifiable or protected information from the research synthesis and publication.

## 3. Results

### 3.1. Evidence Synthesis Outcomes

The multidisciplinary evidence synthesis identified ten major evidence domains that collectively informed the development of the proposed evidence-informed strategic framework for veterinary vaccine manufacturing modernization toward PIC/S GMP compliance. These evidence domains emerged through the multidisciplinary integration of international regulatory guidance, peer-reviewed scientific literature, multidisciplinary feasibility assessment, technical consultations, and implementation experience. Rather than representing isolated findings from individual evidence sources, the identified domains reflect the convergence of scientific, regulatory, technical, organizational, and operational knowledge considered essential for strategic modernization planning.

The identified evidence domains comprised regulatory, technical, organizational, economic, legal, environmental, governance, workforce, infrastructure, and implementation domains. Each domain contributed distinct but complementary perspectives to framework development and collectively provided the conceptual foundation for subsequent framework construction. The multidisciplinary nature of these domains demonstrates that modernization of veterinary vaccine manufacturing extends beyond engineering and regulatory compliance, requiring simultaneous consideration of organizational capacity, financial sustainability, institutional governance, workforce development, and long-term implementation planning.

Among the identified domains, regulatory and technical evidence established the scientific and compliance foundations required for PIC/S GMP implementation, whereas organizational, governance, workforce, and implementation evidence addressed operational readiness and institutional capacity. Economic, legal, and environmental evidence provided additional strategic considerations necessary for sustainable modernization planning. The integration of these complementary evidence domains enabled the development of a holistic framework that reflects both international regulatory expectations and practical implementation requirements while remaining adaptable to diverse institutional contexts.

A summary of the evidence domains identified through the multidisciplinary evidence synthesis is presented in Table 3.

### 3.2. Framework Development Outcomes

The multidisciplinary evidence synthesis was translated into the strategic framework through a traceable evidence-to-framework pathway. Evidence from regulatory and technical documents, scientific literature, multidisciplinary feasibility assessment, and practice-informed implementation experience was first summarized into generalized thematic findings. Related findings were then categorized within the ten evidence domains identified in Section 3.1 and subsequently translated into operational framework components according to their strategic function in veterinary vaccine manufacturing modernization.

This translation demonstrated that individual evidence domains did not operate independently. For example, technical requirements related to vaccine manufacturing were closely connected with infrastructure, regulatory compliance, biosafety, workforce competency, and investment feasibility, while long-term GMP alignment required continuing interaction among governance, organizational capability, quality systems, and implementation processes. Consequently, the framework was structured to preserve these interdependencies rather than treating modernization as a sequence of isolated technical upgrades.

Table 3 provides the evidence-to-framework audit trail, showing the relationship between source evidence, generalized thematic findings, the ten evidence domains, corresponding framework components, and their positioning across the four strategic phases. This mapping formed the basis for subsequent framework refinement and validation.

### 3.3. Framework Refinement and Validation Outcomes

The preliminary framework underwent an iterative refinement process throughout its development to enhance scientific consistency, regulatory alignment, practical applicability, and strategic coherence. Rather than representing a single-stage framework construction process, framework development involved repeated cycles of evidence review, multidisciplinary consultation, and methodological refinement, allowing emerging implementation insights to be systematically incorporated while maintaining consistency with international regulatory standards.

The refinement process improved the framework in four key aspects. First, overlapping implementation activities were consolidated into integrated strategic components, reducing complexity while preserving essential regulatory requirements. Second, cross-cutting elements such as governance, workforce development, and quality management were repositioned as enabling components that support all phases of modernization rather than functioning as independent activities. Third, implementation sequencing was reorganized into a logical progression that better reflects real-world project implementation. Finally, the framework was generalized by removing institution-specific procedures and emphasizing transferable strategic principles applicable across different veterinary vaccine manufacturing settings.

The iterative refinement process resulted in several important improvements to the framework. Individual evidence domains were consolidated into broader strategic components to improve conceptual clarity and reduce redundancy. Logical relationships among regulatory, technical, organizational, governance, and implementation domains were strengthened to better reflect the interdependent nature of vaccine manufacturing modernization. Furthermore, the framework evolved from a collection of thematic recommendations into an integrated strategic model capable of supporting multidisciplinary decision-making throughout the modernization process.

Importantly, refinement focused on strengthening the structure and applicability of the framework rather than modifying project-specific technical findings. Consequently, the final framework represents a generalized strategic planning model that is evidence-informed, operationally relevant, and adaptable to different institutional and regulatory environments while fully respecting confidentiality obligations associated with the consultancy project.

Framework refinement was undertaken through three rounds of multidisciplinary review and technical consultation. The first round supported refinement of the scope and completeness of the evidence domains, particularly where regulatory, technical, economic, organizational, and implementation considerations overlapped. The second round strengthened the translation of these domains into operational framework components and clarified their interdependencies. The third round focused on the overall structure and practical sequencing of the framework, resulting in refinement of the four-phase pathway comprising Design, Construction, Validation, and Continuous Improvement.

Across the consultation rounds, the principal effect of the review process was not the addition of isolated technical requirements, but the progressive strengthening of relationships among framework components. Regulatory requirements were more explicitly connected with facility and process planning; workforce and quality-system considerations were extended across the implementation phases; and economic feasibility, governance, and long-term sustainability were incorporated as continuing considerations rather than discrete pre-construction activities. No quantitative consensus score was calculated because the consultation process was intended for technical and practical refinement rather than statistical validation.

### 3.4. Evidence-Informed Strategic Framework for Veterinary Vaccine Manufacturing Modernization

**Figure 3:**
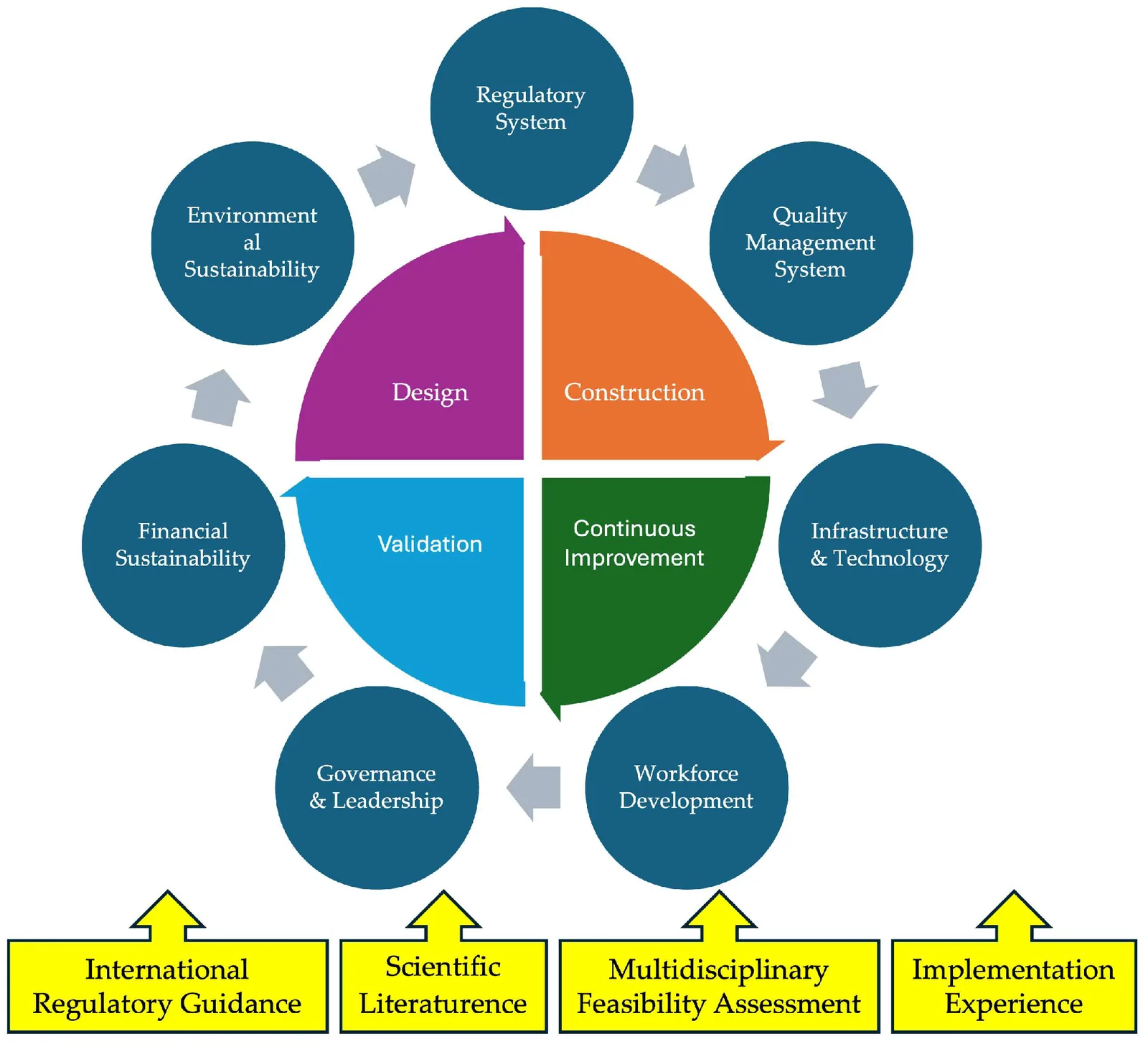
Evidence-informed strategic framework for veterinary vaccine manufacturing modernization toward PIC/S GMP compliance.

The framework development methodology resulted in an evidence-informed strategic framework that integrates multidisciplinary evidence into a structured roadmap for veterinary vaccine manufacturing modernization toward PIC/S GMP compliance (Figure 3). Unlike conventional implementation models that primarily emphasize infrastructure development or regulatory compliance, the proposed framework adopts a systems-based perspective by incorporating regulatory, technical, organizational, financial, legal, environmental, governance, workforce, and implementation dimensions within a single strategic planning model.

The framework is organized into four sequential but interconnected strategic phases: Design, Construction, Validation, and Continuous Improvement. Each phase represents a distinct stage of modernization while maintaining dynamic interactions with the other phases to support adaptive implementation throughout the project lifecycle.

The Design phase establishes the strategic foundation for modernization through regulatory planning, feasibility assessment, investment analysis, organizational preparation, and implementation planning. The Construction phase focuses on translating strategic plans into operational capability through facility development, infrastructure improvement, pharmaceutical quality system implementation, workforce development, and manufacturing readiness. During the Validation phase, manufacturing systems, quality management processes, and regulatory requirements are systematically verified to ensure compliance with PIC/S GMP principles and operational preparedness. Finally, the Continuous Improvement phase emphasizes long-term sustainability through performance monitoring, quality improvement, organizational learning, governance enhancement, and continuous regulatory adaptation.

Although presented as sequential phases, the framework is intended to function as a dynamic management system rather than a linear implementation pathway. Continuous feedback among framework components enables evidence generated during implementation to inform strategic decision-making and ongoing refinement, thereby supporting sustainable modernization under changing regulatory, technological, and organizational conditions.

The principal components of the proposed framework and their strategic functions are summarized in Table 4. A central feature of the final framework was the incorporation of veterinary vaccine-specific requirements within the common modernization pathway. Although the four strategic phases provide a shared structure for modernization, their technical application is not uniform across vaccine products. Requirements are determined by the target animal species, vaccine platform, pathogen characteristics and biological risk, production process, raw-material profile, production scale, and storage and distribution requirements. Consequently, biosafety and biocontainment, process segregation, contamination control, biological raw-material management, and cold-chain assurance were incorporated as cross-cutting technical considerations across the framework.

The Design phase begins with characterization of the intended veterinary vaccine portfolio, including target species, vaccine platform, production organism or biological agent, pathogen risk, manufacturing process, containment requirements, raw-material sources, production scale, and cold-chain requirements. For live-agent vaccines, biosafety and biocontainment considerations are integrated from the outset into facility zoning, process segregation, personnel and material flows, air-handling strategy, decontamination, and waste management.

The Construction phase translates these requirements into appropriately segregated production areas, containment-related engineering controls, utilities, equipment, personnel and material flows, cleaning and disinfection systems, environmental monitoring capability, and biological waste-management infrastructure. Facility implementation is therefore product- and risk-specific rather than based on a uniform engineering model.

The Validation phase includes qualification of facilities, utilities, and equipment; process and cleaning validation; verification of contamination-control and containment measures where applicable; and confirmation of product-specific quality-control systems. Cold-chain qualification, temperature monitoring, storage conditions, transportation controls, and management of temperature excursions are incorporated according to the stability and quality requirements of individual veterinary vaccine products.

The Continuous Improvement phase maintains regulatory compliance and manufacturing capability through deviation and change control, supplier qualification, monitoring of biological and animal-derived raw materials, cold-chain performance review, biosafety and biosecurity reassessment, preventive maintenance, workforce competency development, and periodic review when new vaccine platforms, pathogens, products, or technologies are introduced.

#### Illustrative Hypothetical Application of the Strategic Framework

To demonstrate the practical application of the framework while preserving the confidentiality of the government-commissioned project, a sanitized hypothetical example was constructed. This example does not represent a specific facility or reproduce project-derived operational, engineering, financial, or proprietary information; rather, it illustrates how the framework could guide modernization decisions in an established veterinary vaccine manufacturing facility.

Consider an established facility producing multiple veterinary vaccines that seeks progressive alignment with PIC/S GMP requirements while maintaining continuity of vaccine supply. During the Design phase, the facility would first characterize its vaccine portfolio, manufacturing processes, pathogen-specific biosafety and biocontainment requirements, facility configuration, personnel and material flows, utilities, quality systems, workforce capability, biological raw-material supply, cold-chain requirements, regulatory gaps, and economic feasibility. These findings would be integrated to prioritize modernization requirements and determine whether renovation, partial reconstruction, or replacement of specific production areas represents the most appropriate strategy.

During the Construction phase, prioritized requirements would be translated into facility and engineering modifications, including appropriate production-area segregation, controlled personnel and material flows, containment-related engineering systems, utilities, equipment, environmental monitoring capability, decontamination systems, and biological waste management. Implementation would be sequenced according to regulatory priority, biosafety risk, technical feasibility, available resources, and the need to maintain essential vaccine-production capacity.

During the Validation phase, the upgraded facility, utilities, equipment, processes, and quality systems would undergo appropriate qualification and validation. Critical engineering and containment functions would be verified, personnel competency confirmed, relevant manufacturing and cleaning processes validated, documentation systems assessed, and storage and cold-chain systems qualified before sustained routine operation.

During Continuous Improvement, the facility would maintain compliance and operational readiness through deviation and change control, corrective and preventive actions, supplier and raw-material monitoring, preventive maintenance, workforce competency development, cold-chain performance review, biosafety and biosecurity reassessment, regulatory surveillance, and periodic management review. Introduction of a new vaccine platform, pathogen, manufacturing technology, or major process change would trigger reassessment of relevant framework components and, where appropriate, renewed Design, Construction, or Validation activities.

This hypothetical application illustrates that the framework functions as an iterative modernization pathway rather than a one-time infrastructure-upgrading sequence. The specific technical requirements within each phase must be adapted to the vaccine platform, target species, pathogen risk, manufacturing process, facility context, regulatory environment, and available resources.

### 3.5. Strategic Insights Derived from Framework Development

Framework development and refinement yielded five cross-cutting strategic insights that extended beyond individual technical requirements: (i) renovation versus reconstruction should be determined through integrated technical, regulatory, biosafety, and lifecycle assessment rather than infrastructure condition alone; (ii) workforce capability should be developed concurrently with facility modernization; (iii) public–private partnership may provide an implementation option where technical capability, investment requirements, or long-term operational capacity cannot be adequately supported by a single institution; (iv) operational sustainability should be considered from the Design phase rather than after construction; and (v) long-term governance is required to maintain regulatory alignment, quality systems, institutional accountability, and continuous improvement.

These insights were retained as strategic outputs because they emerged across multiple evidence domains and influenced more than one implementation phase. Table 5 summarizes the relationship between each strategic insight and its corresponding framework implication. Their broader interpretation and relevance to veterinary vaccine manufacturing policy and implementation are discussed in Section 4.

Collectively, these strategic insights demonstrate that veterinary vaccine manufacturing modernization represents a multidisciplinary systems transformation extending beyond infrastructure upgrading alone. The insights highlight the integration of regulatory, technical, organizational, economic, governance, and implementation considerations across the modernization pathway and may provide potentially transferable guidance for future veterinary biological manufacturing initiatives, subject to contextual adaptation (Table 5).

## 4. Discussion

This study developed an evidence-informed strategic framework for veterinary vaccine manufacturing modernization toward PIC/S GMP compliance using a multidisciplinary framework development methodology. Unlike conventional framework studies based primarily on literature review, the proposed framework was derived through the multidisciplinary integration of international regulatory guidance, peer-reviewed scientific literature, multidisciplinary feasibility assessment, and practice-informed implementation experience. This methodological approach enabled the translation of diverse sources of evidence into a coherent strategic framework that addresses the technical, organizational, regulatory, and governance dimensions of vaccine manufacturing modernization.

The discussion focuses on five principal aspects of the framework development process. First, it examines the role of multidisciplinary evidence synthesis as the methodological foundation of framework development. Second, it discusses how multidisciplinary evidence was translated into an evidence-informed strategic framework. Third, it considers the contribution of framework refinement and validation to scientific rigor and practical applicability. Fourth, it explores the broader implications of the proposed framework for veterinary vaccine manufacturing modernization. Finally, it discusses the strengths, limitations, and future applications of the proposed framework.

### 4.1. Multidisciplinary Evidence Synthesis as the Foundation of Framework Development

One of the principal methodological contributions of this study is the adoption of multidisciplinary evidence synthesis as the foundation for framework development. Unlike conventional literature-based framework studies, which rely predominantly on published scientific evidence, the present study integrated international regulatory guidance, peer-reviewed scientific literature, multidisciplinary feasibility assessment, and implementation experience to establish a comprehensive evidence base for veterinary vaccine manufacturing modernization. This approach reflects the complexity of vaccine manufacturing systems, where strategic decision-making extends beyond technical knowledge and requires simultaneous consideration of regulatory, organizational, financial, legal, environmental, and governance dimensions [28,29].

Evidence synthesis has increasingly been recognized as an effective approach for developing implementation frameworks because it enables the integration of multiple forms of evidence rather than privileging a single source of knowledge [28]. In implementation science, practical decision-making often requires combining empirical evidence with contextual knowledge and operational experience to improve the applicability and transferability of implementation strategies [29,30]. The findings of the present study are consistent with this perspective, demonstrating that veterinary vaccine manufacturing modernization should be understood as a multidisciplinary systems transformation rather than a purely technical or engineering process.

Previous studies addressing vaccine manufacturing modernization have generally focused on individual dimensions such as manufacturing technologies [8,9,10,11], production economics [1,2], or technology transfer [11]. Although these studies provide valuable scientific and regulatory knowledge, they are typically presented within discipline-specific perspectives and therefore offer limited guidance for integrating multiple strategic considerations into a unified modernization framework. The present study addresses this gap by synthesizing evidence across multiple disciplines and translating the resulting knowledge into a structured strategic framework applicable to veterinary vaccine manufacturing modernization.

An additional strength of the evidence synthesis approach employed in this study is its incorporation of practice-informed implementation knowledge derived from a real-world modernization project while maintaining strict protection of confidential and proprietary information. Rather than reporting institution-specific findings, the study extracted generalized methodological principles and transferable implementation concepts that could be integrated with published scientific evidence and international regulatory guidance. This approach enhances the external relevance of the framework while ensuring scientific transparency and compliance with contractual confidentiality obligations.

Collectively, these findings suggest that evidence synthesis should be considered not merely as a literature review technique but as a methodological strategy for integrating diverse knowledge sources into evidence-informed strategic decision-making. For complex public-sector modernization initiatives such as veterinary vaccine manufacturing, multidisciplinary evidence synthesis provides a more robust foundation for framework development than reliance on published literature alone.

### 4.2. Translating Evidence into an Evidence-Informed Strategic Framework

The second major contribution of this study lies in demonstrating a traceable process for translating multidisciplinary evidence into an evidence-informed strategic framework. While numerous framework studies describe the final framework itself, relatively few explicitly explain how heterogeneous evidence is systematically synthesized, interpreted, and transformed into strategic decision-support tools [28,29]. By documenting this process, the present study contributes not only a strategic framework but also an adaptable and transferable framework development methodology that may be adapted to other veterinary biological manufacturing contexts.

Framework development in this study was guided by principles of knowledge translation, in which scientific knowledge is transformed into forms that are applicable to policy development, organizational planning, and implementation [29]. Rather than considering regulatory guidance, scientific literature, feasibility assessment, and implementation experience as independent sources of information, these complementary evidence sources were iteratively synthesized to identify common implementation principles and strategic priorities. This evidence-to-framework translation process enabled multidisciplinary knowledge to be converted into a coherent strategic model while preserving scientific rigor and practical relevance.

The framework development process also reflects the principles of implementation science, which emphasize that successful implementation depends on the interaction between evidence, organizational context, implementation processes, and stakeholder engagement rather than on scientific evidence alone [28,31,32,33]. Previous implementation frameworks have consistently demonstrated that effective organizational change requires adaptation of evidence to local contexts while maintaining fidelity to established scientific and regulatory standards [34]. Consistent with these concepts, the proposed framework integrates internationally recognized regulatory principles with generalized implementation knowledge to support strategic decision-making without relying on institution-specific operational procedures.

Another important characteristic of the proposed methodology is its application of systems thinking to veterinary vaccine manufacturing modernization. Vaccine manufacturing is a complex socio-technical system in which regulatory compliance, infrastructure, quality management, workforce capability, governance, financing, and organizational culture interact dynamically throughout the modernization process [11,34]. Consequently, changes in one component inevitably influence the performance of the entire system. By organizing multidisciplinary evidence into interconnected strategic domains rather than isolated technical recommendations, the framework provides a systems-oriented perspective that supports integrated planning and long-term organizational resilience.

Importantly, the framework development methodology adopted in this study emphasizes evidence translation rather than evidence accumulation. The objective was not to compile the largest possible body of evidence but to identify transferable implementation principles capable of supporting strategic modernization across different institutional settings. The incorporation of generalized implementation experience, while maintaining strict confidentiality of project-specific information, further strengthens the practical relevance of the framework without compromising scientific transparency or contractual obligations.

Taken together, these findings suggest that evidence-informed framework development should be regarded as a structured knowledge translation process rather than a conventional literature review exercise. This methodological perspective may provide a useful approach for future studies seeking to develop strategic implementation frameworks in complex regulatory and organizational environments where multidisciplinary evidence must be integrated into practical decision-making.

### 4.3. Framework Validation and Practical Applicability

An important strength of the proposed framework lies in its iterative refinement through expert review, technical consultation, and implementation verification. Rather than relying solely on theoretical assumptions or published evidence, the framework was continuously evaluated against regulatory expectations, multidisciplinary expertise, and practical implementation considerations throughout its development. This iterative refinement process enhanced the scientific credibility and practical applicability of the framework while ensuring consistency with internationally recognized principles of pharmaceutical quality systems.

Framework development studies increasingly recognize that validation extends beyond statistical evaluation and should include assessment of conceptual coherence, scientific consistency, practical feasibility, and implementation relevance [27,33]. In implementation science, expert review and iterative refinement are widely accepted approaches for improving the credibility and usability of strategic frameworks because they enable continuous comparison between theoretical constructs and practical implementation requirements [32]. Consistent with these principles, the framework proposed in this study underwent repeated refinement to strengthen logical relationships among framework components, improve implementation sequencing, and ensure alignment with internationally accepted regulatory standards [35,36].

The incorporation of technical consultation further strengthened the practical applicability of the framework by ensuring that strategic recommendations remained operationally relevant while preserving methodological rigor. Rather than generating recommendations based exclusively on academic evidence, the framework was refined to reflect practical implementation considerations that are frequently underrepresented in published literature. This balance between scientific evidence and implementation knowledge has been identified as a critical factor for successful knowledge translation and implementation of complex organizational interventions [29].

Implementation verification also contributed to strengthening the transferability of the framework. Instead of focusing on institution-specific operational procedures, the refinement process emphasized generalized implementation principles that could be adapted to different veterinary vaccine manufacturing settings. Such an approach is consistent with contemporary implementation science, which recognizes that successful implementation requires adaptation to local organizational contexts while maintaining fidelity to core scientific and regulatory principles [29,33].

Collectively, these findings suggest that framework validation should be viewed as an iterative process of scientific refinement rather than a single verification activity [37]. By integrating expert knowledge, technical consultation, regulatory consistency, and implementation verification, the proposed framework achieved a balance between conceptual robustness and practical applicability [38]. This combination enhances its potential utility as a strategic decision-support tool for veterinary vaccine manufacturing modernization across diverse institutional environments [39].

### 4.4. Strategic Implications for Veterinary Vaccine Manufacturing Modernization

Beyond its methodological contribution, the proposed framework has broader strategic implications for veterinary vaccine manufacturing modernization, particularly for governmental organizations, regulatory authorities, vaccine manufacturers, and low- and middle-income countries (LMICs) seeking to strengthen domestic vaccine production capacity. The framework provides a structured decision-support approach that integrates regulatory compliance with organizational development, investment planning, governance, and implementation strategy, thereby extending its application beyond individual modernization projects.

For governmental agencies, the framework may serve as a strategic planning tool to support national initiatives aimed at strengthening veterinary vaccine manufacturing capacity and improving preparedness for transboundary animal diseases and emerging infectious disease threats. Many countries continue to face challenges in achieving sustainable domestic production of veterinary biological products because modernization requires coordinated policy development, infrastructure investment, regulatory strengthening, and long-term workforce development [1,2,11]. By integrating these multidimensional considerations within a single framework, the proposed methodology may assist governments in prioritizing investments and coordinating modernization activities across multiple sectors.

For vaccine manufacturers, the framework offers a systematic approach to modernization that extends beyond compliance with Good Manufacturing Practice (GMP) requirements. Previous studies have emphasized that sustainable pharmaceutical manufacturing depends not only on technological capability but also on organizational readiness, quality culture, governance, and continuous improvement [8,40,41]. Consistent with these observations, the proposed framework encourages manufacturers to view modernization as a long-term organizational transformation rather than a finite infrastructure project, thereby supporting sustainable regulatory compliance and operational resilience [42,43].

The framework may be particularly relevant for low- and middle-income countries (LMICs), where veterinary vaccine manufacturing capacity is often constrained by limited financial resources, workforce shortages, fragmented regulatory systems, and restricted access to advanced manufacturing technologies [1,2,11]. Because the proposed framework emphasizes strategic planning, phased implementation, and multidisciplinary evidence integration rather than prescriptive technical specifications, it may be adapted to diverse institutional contexts while maintaining alignment with internationally recognized regulatory principles. Such flexibility may facilitate progressive modernization according to local priorities, available resources, and national regulatory capacities.

More broadly, the proposed framework contributes to ongoing international efforts to strengthen resilient and sustainable veterinary vaccine manufacturing systems. Global experiences following recent transboundary animal disease outbreaks and emerging zoonotic threats have highlighted the importance of robust regional manufacturing capacity, regulatory harmonization, and strategic preparedness for biological product production [8,11]. By integrating regulatory science, implementation science, and systems thinking within a unified strategic planning model, the present framework provides a practical methodology that may support evidence-informed policy development and modernization planning beyond the specific context of this study [42].

### 4.5. Strengths, Limitations, and Future Perspectives

This study has several strengths. First, the framework was developed through the integration of multiple complementary evidence sources, including international regulatory and technical guidance, peer-reviewed scientific literature, multidisciplinary feasibility assessment, and practice-informed implementation experience. This approach allowed veterinary vaccine manufacturing modernization to be considered beyond infrastructure upgrading alone and incorporated regulatory, technical, organizational, economic, governance, workforce, and implementation dimensions. Second, the framework-development process was systematically documented through evidence categorization, evidence-to-framework mapping, and multidisciplinary consultation and refinement. Third, the inclusion of practical experience from a government-commissioned modernization project provided an implementation perspective that complemented the scientific and regulatory evidence.

Several limitations should nevertheless be acknowledged. First, the scientific literature component was conducted as a structured evidence search for framework development rather than as a systematic or scoping review. Although multiple databases and predefined relevance criteria were used, the focused and non-systematic nature of the search may have introduced selection bias and may not have captured all relevant evidence. The literature component should therefore be interpreted as one contributing evidence source rather than as an exhaustive synthesis of the veterinary vaccine manufacturing literature.

Second, evidence categorization and translation into framework components involved interpretative judgment. The multidisciplinary nature of the evidence required synthesis across regulatory, technical, organizational, economic, and implementation considerations that could not be reduced to a single quantitative analytical procedure. Although an evidence-to-framework audit trail and three rounds of multidisciplinary consultation were used to improve traceability and reduce dependence on individual interpretation, some degree of subjective judgment remains inherent in the framework-development process.

Third, the framework has not yet been empirically validated across multiple independent veterinary vaccine manufacturing settings. The practice-informed evidence was primarily derived from one government-commissioned modernization context, although that context involved multiple production facilities and vaccine types. Regulatory requirements, institutional arrangements, available resources, vaccine portfolios, biosafety requirements, and manufacturing capabilities may differ substantially across countries and organizations. Accordingly, the proposed framework should be considered potentially transferable rather than universally generalizable, and its application requires adaptation to the specific regulatory, technical, institutional, and resource context.

Fourth, contractual confidentiality and intellectual-property requirements restricted the reporting of project-specific information, including detailed facility assessments, engineering configurations, production capacities, financial analyses, and implementation decisions. Anonymized contextual information and a de-identified application example were therefore provided to demonstrate how practice-informed evidence contributed to framework development without disclosing protected information. Nevertheless, these restrictions limit independent examination of some of the underlying practical evidence.

Future research should therefore focus on prospective application and external validation of the framework across multiple veterinary vaccine manufacturing settings. Evaluation across different vaccine platforms, animal species, pathogen-risk and containment requirements, facility scales, regulatory environments, and resource settings would help determine which framework components are broadly transferable and which require contextual modification. Future studies should also develop measurable implementation indicators to evaluate regulatory progress, technical readiness, workforce capability, operational performance, investment requirements, and long-term sustainability. Comparative multicenter application and longitudinal evaluation would provide stronger empirical evidence for subsequent refinement of the framework.

## 5. Conclusions

This study developed an evidence-informed strategic framework for veterinary vaccine manufacturing modernization toward PIC/S GMP compliance through a transparent framework development methodology integrating multidisciplinary evidence synthesis, regulatory science, implementation science, and practice-informed implementation experience. Unlike conventional framework studies that rely primarily on literature review or discipline-specific evidence, the proposed methodology systematically translated international regulatory guidance, peer-reviewed scientific literature, multidisciplinary feasibility assessment, and generalized implementation knowledge into a coherent strategic framework capable of supporting complex modernization initiatives.

The findings demonstrate that veterinary vaccine manufacturing modernization should not be regarded solely as an infrastructure or engineering project, but rather as a multidisciplinary organizational transformation requiring coordinated development of regulatory systems, pharmaceutical quality management, infrastructure, workforce capability, governance, financial sustainability, and long-term implementation strategies. By adopting systems thinking and evidence-informed decision-making principles, the proposed framework provides a comprehensive approach for integrating these interdependent dimensions into strategic modernization planning.

Importantly, the principal contribution of this study extends beyond the framework itself. The study demonstrates a transparent and systematically documented and traceable methodology for translating diverse evidence sources into strategic decision-support tools while maintaining scientific rigor, practical applicability, and protection of confidential information generated during government-commissioned consultancy projects. This methodological contribution may be applicable not only to veterinary vaccine manufacturing modernization but also to other complex regulatory and organizational transformation initiatives involving multidisciplinary evidence integration.

Future studies should evaluate the implementation of the proposed framework across different veterinary biological manufacturing organizations and regulatory environments to further examine its transferability, implementation outcomes, and long-term organizational impact. Such research would strengthen the evidence base for framework-guided modernization and support the development of resilient, sustainable, and internationally competitive veterinary vaccine manufacturing systems.

Ultimately, this study provides both a strategic framework and a transparent framework development methodology that may assist governments, regulatory authorities, vaccine manufacturers, and researchers in designing evidence-informed modernization strategies capable of strengthening veterinary biological manufacturing capacity while supporting global animal health, One Health preparedness, and sustainable vaccine production.

## Figures and Tables

**Table 1:** Evidence domains incorporated into the framework development methodology.

Evidence Source	Purpose	Examples	Contribution to Framework Development
International regulatory and technical guidance	Establish regulatory principles	PIC/S, WHO, EMA, FDA	Regulatory requirements and GMP principles
Peer-reviewed scientific literature	Provide scientific evidence	Vaccine, Biologicals, Expert Review of Vaccines	Best practices, implementation concepts, critical success factors
Multidisciplinary feasibility assessment	Generate practical evidence	Technical, economic, legal, environmental, and organizational assessments	Context-specific implementation considerations
Technical consultation and implementation experience	Refine framework applicability	Government experts, academia, industry, veterinary professionals	Practical validation and framework refinement

**Table 2:** Framework validation criteria applied during framework refinement.

Validation Domain	Purpose	Evidence Considered
Scientific consistency	Ensure alignment with published evidence	Peer-reviewed literature
Regulatory consistency	Verify compliance with international GMP principles	PIC/S, WHO, EMA, FDA
Technical feasibility	Assess practical implementation	Technical feasibility assessment
Organizational applicability	Evaluate implementation readiness	Expert consultation
Strategic relevance	Assess usefulness for modernization planning	Government and industry consultation

**Table 3:** Evidence-to-Framework Audit Trail and Translation of Evidence Domains into Framework Components.

Generalized Thematic Finding	Evidence Domain	Framework Component	Strategic Phase(s)
International regulatory and technical guidance; scientific literature; practice-informed evidence	Modernization requires progressive alignment of manufacturing systems, documentation, quality processes, and regulatory expectations with internationally recognized GMP requirements.	Regulatory	Regulatory alignment, GMP gap assessment, compliance planning, and regulatory readiness
Scientific literature; technical standards; feasibility assessment; implementation experience	Veterinary vaccine manufacturing requires product- and process-specific consideration of production technology, process flow, segregation, contamination control, utilities, and biosafety requirements.	Technical	Manufacturing-process assessment, technology requirements, biosafety/biosecurity integration, and technical modernization planning
Feasibility assessment; implementation experience	Successful modernization requires coordination among technical units, quality functions, management, procurement, finance, and operational responsibilities rather than isolated infrastructure development.	Organizational	Organizational readiness, cross-functional coordination, responsibility allocation, and change management
Feasibility assessment; economic evidence; implementation experience	Capital investment decisions must account for implementation priorities, lifecycle costs, operational sustainability, and the financial consequences of alternative modernization pathways.	Economic	Economic feasibility assessment, investment prioritization, financing strategy, and long-term sustainability planning
Regulatory evidence; feasibility assessment; government-project context	Modernization decisions are constrained by applicable legal mandates, procurement requirements, institutional authority, contractual arrangements, and potential public–private implementation models.	Legal	Legal and institutional assessment, procurement considerations, contractual planning, and PPP feasibility
Regulatory and technical guidance; feasibility assessment	Facility modernization may affect utilities, waste management, biological waste handling, energy demand, environmental controls, and long-term resource requirements.	Environmental	Environmental compliance, waste-management planning, utility sustainability, and environmental risk consideration
Regulatory evidence; feasibility assessment; implementation experience	Sustained GMP alignment depends on clear accountability, decision-making authority, quality oversight, resource allocation, and mechanisms for maintaining institutional commitment over time.	Governance	Governance structure, accountability, quality oversight, strategic decision-making, and institutional continuity
Regulatory guidance; feasibility assessment; implementation experience	Modernized facilities require personnel with appropriate GMP knowledge, technical competencies, quality-system capability, biosafety awareness, and continuing professional development.	Workforce	Workforce planning, competency assessment, GMP training, technical capacity building, and competency maintenance
Technical guidance; feasibility assessment; practice-informed evidence	Existing buildings, process layouts, utilities, containment systems, equipment, and supporting infrastructure may limit the feasibility and sequencing of modernization.	Infrastructure	Facility assessment, engineering gap analysis, utilities and equipment planning, containment and segregation requirements, and phased infrastructure upgrading
All four evidence sources	Modernization is an iterative institutional process requiring prioritization, sequencing, verification, adaptation, and continued performance monitoring rather than a one-time construction project.	Implementation	Phased implementation, risk-based prioritization, implementation monitoring, verification, adaptive management, and continuous improvement

Note: Evidence domains were not derived from individual evidence sources in isolation. They represent categories generated through multidisciplinary integration of regulatory and technical documents, scientific literature, feasibility assessment, and practice-informed implementation experience. Project-derived findings are presented only at a generalized level to preserve contractual confidentiality and intellectual property.

**Table 4:** Principal components of the evidence-informed strategic framework.

Strategic Insight	Framework Implication	Strategic Insight
Renovation vs. reconstruction	Requires integrated assessment during Design before major capital commitment	Renovation vs. reconstruction
Workforce capability	Must progress concurrently with technical and infrastructure modernization	Workforce capability
Public–private partnership	Provides a potential implementation mechanism where capability or investment gaps exist	Public–private partnership
Operational sustainability	Must be incorporated from Design through Continuous Improvement	Operational sustainability
Long-term governance	Maintains accountability, regulatory alignment, quality systems, and institutional continuity	Long-term governance

**Table 5:** Practical and framework implications of strategic insights identified during framework development.

Strategic Insight	Practical Implication	Framework Implication
Modernization extends beyond facility renovation	Modernization requires coordinated organizational transformation	Infrastructure is one component of a multidisciplinary modernization strategy
Workforce capability is a strategic enabler	Continuous competency development and leadership commitment are essential	Workforce development supports all phases of implementation
Integrated governance enhances implementation	Coordination among regulatory agencies, academia, and industry improves implementation effectiveness	Governance functions as a cross-cutting framework component
Sustainable financing supports long-term modernization	Phased investment and public–private collaboration strengthen implementation feasibility	Financial sustainability is embedded throughout the modernization lifecycle
Continuous improvement ensures long-term resilience	Ongoing monitoring, quality improvement, and organizational learning sustain GMP compliance	Modernization is represented as a continuous strategic management process

## Data Availability

Relevant information is included in the article.
